# Mapping of cholera Priority Areas for Multisectoral Interventions (PAMIs) using the 2023 GTFCC guidance: The Kenya experience

**DOI:** 10.1371/journal.pntd.0013078

**Published:** 2025-06-20

**Authors:** Catherine Kiama, Emmanuel Okunga, Catherine Makwe, Annastacia Muange, Martins Livinus, Daniel Langat, Joan Brunkard, Anne Loarec

**Affiliations:** 1 Washington State University – Global Health, Nairobi, Kenya; 2 Division of Disease Surveillance and Response, Ministry of Health, Nairobi, Kenya; 3 Country support Platform, International Federation of Red Cross and Red Crescent Societies (IFRC), Brazzaville, Congo; 4 World Health Organization, Kenya Country Office, Nairobi, Kenya; 5 US Centers for Disease Control and Prevention, Atlanta, Georgia, United States of America; 6 Consultant, International Federation of Red Cross and Red Crescent Societies (IFRC), Brazzaville, Congo; University of Connecticut, UNITED STATES OF AMERICA

## Abstract

Cholera Priority Areas for Multisectoral Interventions (PAMIs), formerly known as “hotspots”, are limited geographical areas where cholera persists or regularly reappears due to cultural, environmental, and socioeconomic conditions. Focusing interventions on PAMIs will help to effectively control and ultimately eliminate cholera among the most at-risk populations. The 2023 GTFCC Methodology was used to identify PAMIs for cholera control in Kenya. The analysis was conducted between February and March 2024, selecting PAMIs based on the previous six years’ epidemiological data (Jan 2018 - Dec 2023) at the sub-county level. Epidemiological data was sourced from cholera outbreak line lists. The line list included both confirmed and suspected cholera cases of all ages admitted or reported to health facilities. The numerical priority index was calculated as a sum of four epidemiological indicators: incidence, mortality, persistence, and laboratory testing. Following a validation workshop, stakeholders selected a priority index threshold, identifying 78 sub-counties as initial PAMIs. There were 29 additional PAMIs included in the final list of 107 priority sub-counties based on country-specific vulnerability factors. This evidence-based approach will inform the targeting and implementation of multi-sectoral interventions in line with the Kenya National Cholera Plan.

## Author summary

Kenya continues to experience cholera outbreaks, particularly in areas with limited access to safe water, sanitary facilities, and health services. In response, Kenya has implemented the 2023 Global Task Force on Cholera Control (GTFCC) method to identify the Priority Areas for Multisectoral Interventions (PAMIs). By analyzing six years of national data (2018–2023), we calculated a priority index score based on four key epidemiological indicators: cholera incidence, mortality, persistence of outbreaks, and laboratory testing capacity. The data were analyzed at the subcounty administrative level for each of the 304 subcounties.

A team of subnational and national stakeholders identified 78 sub-counties as PAMIs due to high-priority index scores. Additionally, 29 sub-counties were included as PAMIs due to evidence of local vulnerability factors. In total, the 107 PAMIs encompass 40% of Kenya’s population and account for over 97% of the reported cholera cases and deaths.

This targeted approach ensures that specific interventions, such as expansion of water and sanitation infrastructure and preventive vaccination campaigns, are directed to the areas that need them most. Kenya’s experience serves as a model for other countries by demonstrating the importance of inclusive, data-driven prioritization of intervention areas, especially where resources, such as vaccine supplies, are limited.

## Introduction

Cholera remains a significant public health concern, with 1.3- 4 million cases and 21,000 – 143,000 deaths reported globally each year [1]. WHO noted a global resurgence of cholera since 2021. Over 61,000 cases and 3,500 deaths were recorded in 2023, surpassing those in 2022, with 44 countries reporting cases compared to 35 in 2021. WHO designated the cholera resurgence as a grade 3 emergency. The resurgence adversely affected Africa, with 16 African countries reporting cases. The recent outbreaks have recorded the highest case-fatality ratios in over a decade, leading to a shortage of resources and supplies for an effective response [2,3].

Kenya reported its first case of cholera in 1971 and continues to experience cyclical outbreaks every few years [2]. The country recorded over 12,000 cases in 2022 – 2023, with a case fatality ratio of 1.7%, affecting more than half of its 47 counties [2]. The outbreak has continued to affect different parts of the country in 2024, worsened by the flash floods and massive displacement of persons witnessed during the March to May unusually heavy rains. Over 290,000 persons were displaced by floods across the country [4]. Kenya deployed response measures, including mass oral cholera vaccination campaigns in the most affected areas in 2023, where over 3 million persons received a single dose of the oral cholera vaccine (OCV) amidst a global vaccine shortage [5,6].

The cholera situation is part of a larger problem in Kenya. The burden of infectious and non-communicable diseases in Kenya is increasing due to socioeconomic disparities, inadequate healthcare infrastructure, and environmental challenges [7,8]. The Global Roadmap to End Cholera by 2030 calls for control and elimination strategies spatially targeted to Priority Areas for Multisectoral Interventions (PAMIs) [9]. Countries develop a National Cholera Plan (NCP) to guide the implementation of interventions across all pillars to control cholera in the priority areas. Identification of PAMIs is an essential step in the NCP development. In 2023, the Global Task Force on Cholera Control (GTFCC) developed guidance and tools to guide countries in identifying PAMIs for cholera control [10]. In countries with high to moderate cholera transmission, identifying PAMIs to control cholera helps understand the spatial distribution of the cholera burden at the subnational level enabling targeted control strategies to ultimately reduce morbidity and mortality [10].

In 2021, Kenya conducted a hotspot mapping exercise to identify priority areas as part of the NCP development [11–13]. The hotspot mapping focused on five years of surveillance data from 2015 to 2019, based on the GTFCC guidance available at that time. With the global resurgence of cholera in recent years, the epidemiological situation has changed, with unmapped areas affected during the prolonged 2022–2024 outbreak. Unpredictable climate events, such as heavy rains and flooding, disrupted the Water, Sanitation, and Hygiene (WaSH) infrastructure with massive population displacement. Significant population movement across international borders during the conflict and migration to urban towns and cities for economic reasons were also observed. Considering the changes in cholera dynamics, Kenya applied the new GTFCC method to guide the NCP interventions and ensure the optimization of resources to maximize impact.

Our paper provides the results of utilizing the updated GTFCC method to identify PAMIs for cholera control as applied in the Kenyan context. We present insights into how surveillance and laboratory testing data, vulnerability factors, and stakeholder consultation at national and subnational levels were used to identify the PAMIs. Kenya is one of the first countries to use the new methodology to update the priority areas. This paper will be useful for other countries as they adapt the methodology to their local context and plan to develop their NCPs.

## Materials and methods

### Ethics statement

Ethical review was waived since the PAMI identification was based on retrospective data. Access to surveillance data was approved by the Ministry of Health, Kenya. Patient confidentiality was strictly maintained through anonymizing data at analysis with minimal data sharing. This activity was reviewed by the CDC, deemed not research, and was conducted consistent with applicable federal law and CDC policy.

The identification of PAMIs was based on retrospective analysis of routinely collected individual-level cholera data from Jan 2018 to Dec 2023, sourced from the Ministry of Health. In this paper, cholera cases refer to all cholera cases (whether confirmed, probable, or suspected) who were admitted to or reported to the health facilities within the respective sub-counties. Confirmed cases included all cases with laboratory confirmation by culture, probable cases had a positive RDT or were epidemiologically linked to a confirmed case while Suspected cases were those that fit the clinical standard case definition of having acute watery diarrhea with or without severe dehydration or any person dying of acute watery diarrhea. The data variables collected for the analysis included demographic variables (age, gender, location of residence), clinical information (date of admission), laboratory testing data, and patient outcomes.

The GTFCC method was used to identify priority areas for cholera control in Kenya [10]. The country is divided into 47 counties and further into 304 sub-counties. The analysis was at the sub-county level, the lowest administrative level where both the surveillance and population data are available. The interventions outlined in the NCP are to be implemented at the sub-county level. The past hotspot mapping using the 2019 GTFCC method highlighted the importance of using a lower administrative level (sub-county) as the unit of analysis as opposed to a higher administrative level (county). It was observed that there were disparities in cholera burden and risk with large areas included in the county-level analysis, yet not all sub-counties in all counties had high cholera burden [13].

We extracted population estimates for each geographic unit (i.e., sub-county) from the Kenya Health Information System (KHIS). These estimates are projections from the 2019 census for the Kenyan population and the UNHCR data for refugees and asylum seekers [14]. The refugee and asylum seeker populations were combined with the host community population in the respective sub-counties.

For cholera outbreak reporting, individual data is entered into a Microsoft Excel line list for each sub-county. The cholera line lists had missing data. Entries were excluded from the PAMI analysis if the date of admission and location of residence were missing. For other variables, the missing data were considered as zero values, e.g., test variables were considered not tested, and missing outcomes were considered alive. Data cleaning and preparation were performed using R statistical software (v4.3.2; R Core Team 2024), and aggregated data were entered in the GTFCC Excel-based tool in a specific format using the Data model template [10].

Data analysis was conducted using R statistical software (v4.3.2; R Core Team 2024), while QGIS (QGIS.org version 3.34) was utilized to visualize the geographic distribution of the results. Descriptive analysis was performed to summarize frequencies and proportions for categorical variables. The data were aggregated per year and unit (number of cases, number of deaths, number of weeks with cases, number tested by either Rapid Diagnostic Test (RDT) or culture, number positive by RDT or culture). After consistency and format checks, the aggregated data were input into the GTFCC Excel tool, automatically calculating each unit’s epidemiological and testing indicators.

We focused the PAMI identification on four indicators, which were defined as follows over the six years: 1) Incidence: total number of cholera cases per 100,000 person-years 2) Mortality: total number of deaths attributed to cholera reported per 100,000 person-years 3) Persistence: percentage of weeks with at least one cholera case among the total number of weeks 4) Number of years with case(s) tested positive: number of years with at least one case tested positive for cholera (regardless of the testing method). This testing indicator (i.e., number of years with case(s) tested positive) was selected considering the sub-optimal representativeness of testing. Only 71.9% of the surveillance units had a weekly testing coverage ≥ 50%. Weekly testing coverage was calculated as the percentage of weeks with at least one suspected case tested for cholera (by either RDT or culture) among weeks with at least one suspected case. Weekly testing coverage was not applied in the priority index scoring since the threshold of 80% for representativeness was not met.

We calculated the median and the 80th percentile for each of the three epidemiological indicators after excluding the units with zero reported cases. Based on the median and 80th percentile, each indicator was scored, between 0–3 for each unit. For the testing indicator, the number of years with positive tested cases was calculated and scored between 0 and 2 due to the suboptimal representativeness of the weekly testing coverage. The tool automatically calculated the scores for each indicator per unit. The priority index (sum of the four indicator scores) for each unit was also calculated for each administrative unit, per the GTFCC 2023 guidance. The priority index (possible score of 0–11) was used to rank the administrative units.

During an in-person stakeholder workshop in February 2024, the stakeholders defined a priority index threshold of 6. This threshold is set up to balance between the feasibility of the interventions (number of units and % of population targeted) versus the impact on cholera transmission (% of cases and deaths reported in these units). The stakeholders discussed the GTFCC indicative list of vulnerability factors to select factors applicable to Kenya and where data were available at the subnational level. The stakeholders adopted five vulnerability factors to identify the additional PAMIs: areas bordering identified cholera hotspots in a neighboring country, high population density, presence of at-risk populations (refugees, fishermen, and mining populations), access to improved water sources, and access to improved sanitation services. The final list of PAMI included both the initial PAMIs based on the calculated priority index score and additional PAMIs based on the vulnerability factors. The final list of PAMI was discussed and validated during the February 2024 workshop with representatives of national and subnational governments and partner organizations.

## Results

**Fig 1:**
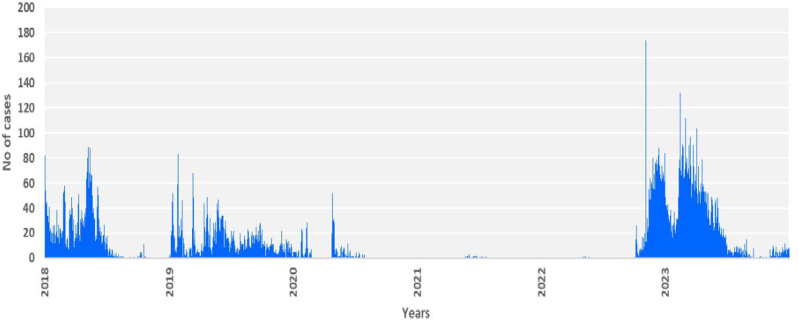
Weekly distribution of cholera cases, 2018 -2023, Kenya.

**Fig 2:**
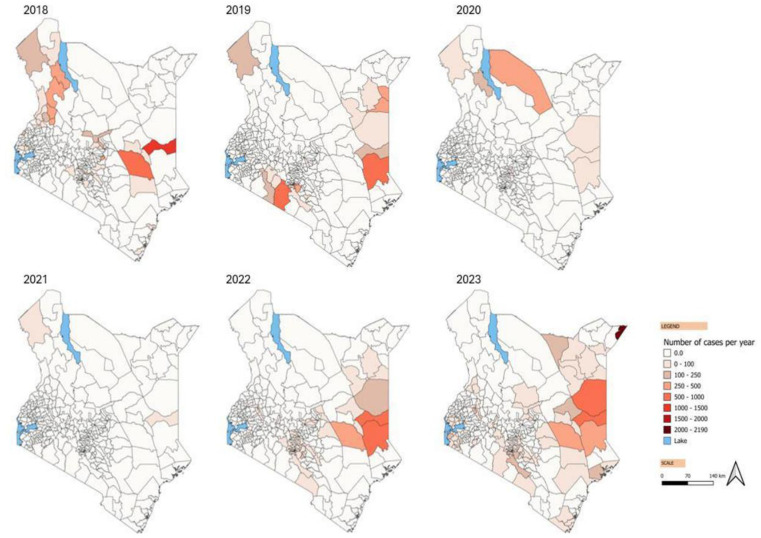
Cholera cases per sub-county and year, 2018 - 2023, Kenya. Maps created on QGIS using OCHA shapefile (source IEBC), Kenya - Subnational Administrative Boundaries - Humanitarian Data Exchange. Accessed March 14, 2024. https://data.humdata.org/dataset/cod-ab-ken?.

The country reported cholera cases in each of the six years, with outbreaks affecting different parts of the country each year. Notably, fewer cholera cases were reported during the COVID-19 pandemic period (Figs 1 and 2).

Between 2018 and 2023, 23,946 cases and 334 deaths were recorded. Only one case was excluded due to missing key variables. Close to half of the sub-counties (151 of 304) reported zero cholera cases in that period (Table 1). Among the 153 sub-counties with at least one case, the median and the 80th percentile of the incidence were 4.04 and 16.20 per 100,000 persons-year, respectively. The median mortality rate was 0.24 per 100,000 persons-year with an 80th percentile of 0.47 per 100,000 persons-year. The median and the 80th percentile for the weekly percentage persistence were 2.2% and 8.5%, respectively. Regarding the testing indicator for the number of years with cases tested positive, there was no testing (score of zero) in 49.7% (n = 151) sub-counties, one year with a positive case (score of 1) in 24.7% (n = 75), while more than one year of positive cases (score of 2) in 25.7% (n = 78) sub-counties.

**Table 1:** Distribution of sub-counties by Priority Index Score, population sizes, cases, and deaths, 2018-2023, Kenya.

Priority index score	No. of sub-counties	Cumulative sub-counties	Population 2023	Cumulative% population	Sum of Cases	Cumulative % Cases	Sum of Deaths	Cumulative % deaths
11	7	7	1,582,411	2.9	5,255	21.9	94	28.1
10	5	12	1,663,662	6.0	6,779	50.3	47	42.2
9	15	27	3,347,372	12.2	4,426	68.7	87	68.3
8	16	43	3,526,497	18.7	3,388	82.9	46	82.0
7	17	60	3,538,238	25.3	1,688	89.9	19	87.7
6	18	78	3,252,674	31.3	1,427	95.9	26	95.5
5	15	93	2,936,073	36.8	482	97.9	8	97.9
4	20	113	3,890,244	44.0	318	99.2	7	100.0
3	40	153	7,020,808	57.0	183	100.0	0	100.0
0	151	304	23,234,404	100.0	0	100.0	0	100.0
Total	304		53,992,383		23,946		334	

Stakeholders considered areas with a priority index value equal to or above six as PAMI. This approach identified 78 (25.7%) initial priority areas with a total population of 16,910,854 (31.3%) and included 95.9% and 95.5% of the reported cholera cases and cholera-related deaths, respectively, during the six years. The distribution of the units per priority index scores is shown in Table 1 with the corresponding population, cases, and deaths.

Stakeholders agreed that the cholera burden in some sub-counties could have been underestimated due to barriers to accessing health facilities in conflict-affected areas and, in some instances, where patients seek care outside their sub-counties of residence. Furthermore, the limited availability of laboratory testing and diagnostic resources with no confirmation of cases contributed to lower priority scores in the hard-to-reach areas. Of the remaining 226 sub-counties, 29 sub-counties with a priority index below six were selected by stakeholders as additional PAMIs due to biased epidemiological indicators and documented vulnerability factors in these areas. The final list of PAMIs represents 107 sub-counties with a total population of 21,574,063 (40% of the 2023 estimated population) and covers 97.3% of the reported cholera cases and 96.4% of cholera-related deaths during the studied period (Table 2). The balance between the feasibility of the intervention and public health impact was discussed to finalize the number and list of PAMIs.

**Table 2:** Distribution of final PAMI sub-counties by population size, cases, and deaths, 2018-2023, Kenya.

^*^Final PAMI decision	Priority index score	No. of sub-counties	^**^Cumulative % sub-counties	Population 2023	% Population	Sum of cases	% cases	Sum of deaths	% deaths
Initial	11	7	2.3	1,582,411	2.9	5,255	21.9	94	28.1
Initial	10	5	3.9	1,663,662	6.0	6,779	50.3	47	42.2
Initial	9	15	8.9	3,347,372	12.2	4,426	68.7	87	68.3
Initial	8	16	14.1	3,526,497	18.7	3,388	82.9	46	82.0
Initial	7	17	19.7	3,538,238	25.3	1,688	89.9	19	87.7
Initial	6	18	25.7	3,252,674	31.3	1,427	95.9	26	95.5
Additional	5	5	27.3	982,013	33.1	184	96.7	8	96.1
Additional	4	6	29.3	1,098,418	35.2	87	97.0	2	96.4
Additional	3	6	31.3	805,753	36.7	67	97.3	1	96.4
Additional	0	12	35.2	1,777,025	40.0	0	97.3	0	96.4
Total		107		21,574,063		23,301		322	

* Initial = Initial PAMI based on a priority index of 6 and above, Additional = Additional PAMI.

** number of total sub-counties = 304.

**Fig 3:**
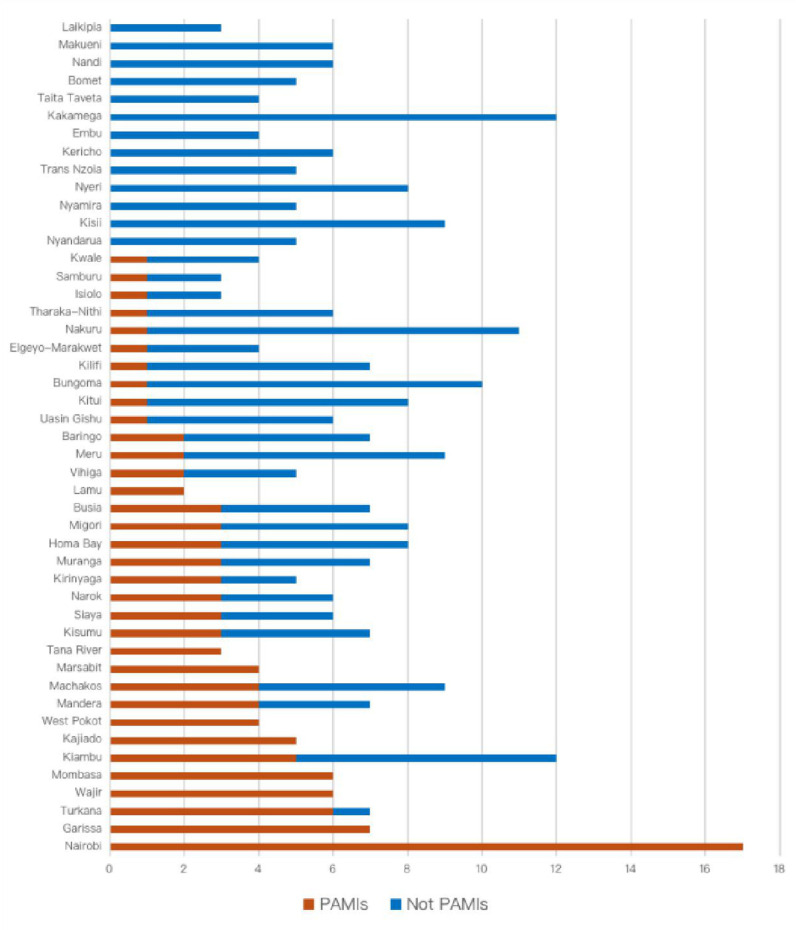
Distribution of the sub-counties by final PAMI decision, stratified by county, Kenya 2024.

**Fig 4:**
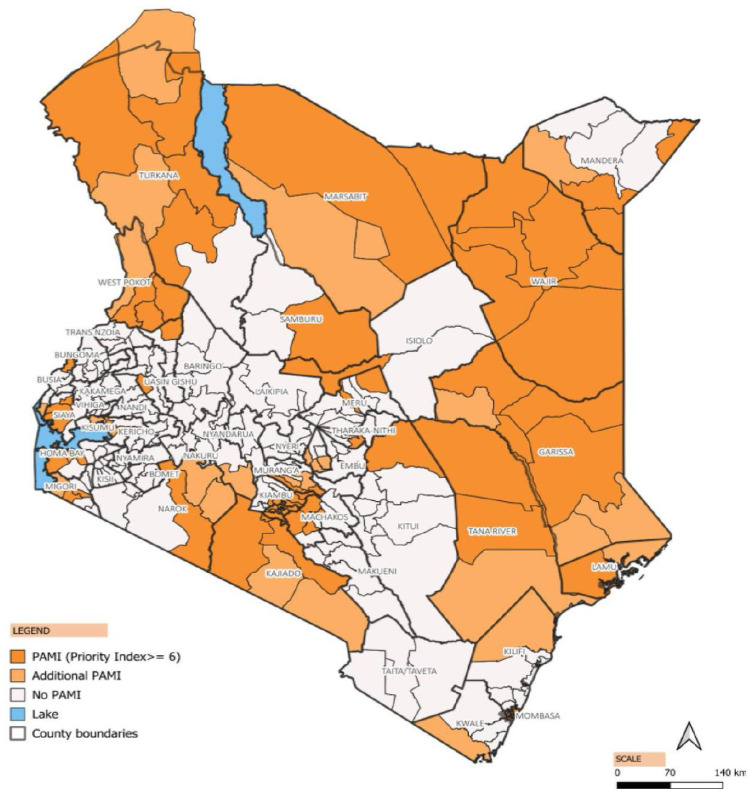
Map of Kenya showing the final PAMIs, 2024. Maps created on QGIS using OCHA shapefile (source IEBC), Kenya - Subnational Administrative Boundaries - Humanitarian Data Exchange. Accessed March 14, 2024. https://data.humdata.org/dataset/cod-ab-ken?.

Nine (out of 47) counties had 100% of their sub-counties considered as PAMIs in the final list, 13 counties had no PAMIs, while 25 counties had at least one or more of their sub-counties considered as PAMI (Figs 3 and 4).

## Discussion

Cholera has been a persistent challenge in Kenya, with cases and deaths reported across each of the six years included in this study. However, there was substantial geographical variability in the cholera burden across sub-counties, with nearly half of the sub-counties reporting zero cholera cases during the period under review. Notably, there were few cases reported in 2020 and 2021. The highest number of cases occurred during the widespread outbreak from 2022 to 2023, which affected 28 counties. Out of the 34 counties with cases over the six years, nine counties had all their sub-counties considered priority areas, while 25 had one or more sub-counties considered. The counties considered as PAMI in entirety are more vulnerable particularly large urban cities, areas located along the Indian Ocean, and areas sharing international borders with other cholera-affected countries. The geographical variability emphasizes the need for localized interventions at the subnational level, considering local risk factors such as water and sanitation conditions, population density, and socioeconomic factors contributing to the population’s vulnerability to cholera. There were notable variations in incidence and persistence over time. Extreme climate events, population movements, conflict, and the COVID-19 pandemic are likely to have influenced the cholera dynamics in recent times.

Kenya has laboratory capacity, with most sub-counties conducting testing using either RDT or culture. However, only 25% of the reporting sub-counties detected at least one positive case in any of the years, suggesting limited access to testing or diagnostic challenges with potential underreporting in remote and rural areas of the country. The capacity of healthcare workers to diagnose and confirm cholera through testing, as well as strengthening reporting mechanisms, could support early detection and timely response to outbreaks.

The PAMI process in Kenya involved extensive stakeholder engagement across various sectors such as health, water, sanitation, subnational teams, and partner organizations. This inclusive approach ensured that diverse perspectives were considered, resulting in a more comprehensive list of priority areas. Wide stakeholder engagement fosters ownership and commitment to facilitate and sustain interventions. Application of the local knowledge and expertise of the sectors played a crucial role in refining the area-specific vulnerability factors.

Stakeholders agreed on a priority index of 6 or higher to designate a sub-county as a PAMI, resulting in 78 initial priority areas, i.e., approximately 30% of Kenya’s population and accounting for over 95% of cholera cases and deaths. The identified initial priority areas largely include sub-counties along international borders with cholera-affected countries, densely populated areas in urban towns, and those along water bodies. Previous hotspot mapping shared similar findings, though these were conducted using fewer indicators per the 2019 GTFCC method [11,13]. Sub-counties that share international borders were primarily identified as initial PAMI (Mandera, Garissa, Wajir, Marsabit, Turkana, Kajiado, Migori, West Pokot, and Busia), suggesting the possibility of cross-border transmission through the refugee camps and asylum seekers. There is a need for cross-border information sharing between neighboring countries, especially during conflict and other humanitarian emergencies. The population movement of persons across international borders makes the case for synchronization of preparedness and response activities such as training, community engagement, and timing of preventive mass vaccination campaigns. Densely populated sub-counties in the urban cities and towns, such as the entire Nairobi and parts of Mombasa, were also featured as priority areas. Urban towns have seen an influx of people from rural areas for economic reasons without water and sanitation infrastructure expansion. Areas with special populations, such as the fishing communities around the water bodies (Lake Victoria, Lake Turkana, Indian Ocean), have experienced a significant influx of people for economic reasons, leading to the expansion of small villages with limited water sources and sanitation facilities. The densely populated areas in the informal urban settlements and communities alongside water bodies should be prioritized for water and sanitation infrastructure expansion to cater to the growing population.

It is optional for countries to conduct the vulnerability assessment using the GTFCC indicative list of 12 vulnerability factors, which include proximity to cholera-affected areas/identified PAMIs, major travel routes, high population density or overcrowded settings, high-risk populations, hard-to-access populations, received oral cholera vaccine more than three years ago, extreme climate and weather conditions, complex humanitarian emergencies, and inadequate access to WaSH. Open defecation, population growth in informal settlements and urban areas without adequate access to drinking water and sanitation infrastructure, cross-border movement of persons from neighboring countries experiencing complex humanitarian crises and large cholera outbreaks, crowded settings such as refugee camps and displaced person camps, conflict due to political instability, mass gathering events and changes in rainfall patterns are some of the risk factors associated with cholera outbreaks in Kenya [2,11,15–17]. An additional 29 sub-counties, with a priority index below 6, were included as PAMIs following the vulnerability assessment, expanding to 107 sub-counties and representing 40% of the population.

Stakeholders made the following considerations applicable to the local context and supported by historical events: a) areas that are surrounded by index PAMIs and along transport corridors, b) recent changes in the administrative boundaries impacting the cholera case reporting in the newly curved sub-counties, c) under-reporting of cholera cases in sub-counties where conflict and insecurity have affected surveillance activities and rendered health facilities inaccessible, d) communities seeking care in neighboring sub-counties away from their places of residence, e) early 2024 sub-county cholera cases not captured by the study period, and finally, f) areas prone to adverse weather events (heavy rains, floods, droughts). The choice of factors 1,4,5,10, and 11 from the GTFCC indicative list and the applied definition were based on published studies conducted in Kenya reporting the most common risk factors during cholera outbreaks.

The PAMI identification offers updated and more robust evidence and risk-based prioritization of the sub-counties compared to previous hotspot mapping versions in the current National Cholera Plan [11,13]. The priority areas cover a large proportion (40%) of the population hence, the national government should consider local contextual factors that increase the risk of local cholera transmission. Knowledge of the local contextual factors can be applied to further prioritize the different regions for specific interventions. Targeted interventions across all pillars of cholera control (including WaSH, preventive OCV, behavior change communication, enhanced surveillance, and laboratory capacity) can improve the effectiveness of the interventions with good use of limited resources. Additionally, understanding the local context can guide specific innovative strategies for vulnerable high-risk areas and indicate when to adjust strategies.

## Conclusion

The PAMI findings are crucial in shaping public health policy in Kenya. The findings support the need to allocate resources and interventions based on risk, to reduce the burden of cholera, and to prevent cholera-related deaths in line with the Ending Cholera Global Roadmap to 2030 [9]. For sustainable progress in cholera control and prevention, the National Cholera Plan must advocate for all actors to focus on the identified PAMIs across all cholera control pillars. It is recommended that countries update the PAMIs every five years to consider available resources and the changing epidemiological and WASH situation. In the future, these findings can help prioritize high-burden areas for interventions, such as expanding access to water and sanitation services, preventive vaccination, and prepositioning of RDTs.

The GTFCC methodology supports data-driven decision-making, using the numeric priority index based on the sum of the epidemiological and testing indicators. The second strong aspect of the methodology is the engagement of stakeholders in the decision-making process. Stakeholders are engaged from setting the priority index threshold to the application of the vulnerability factors and finalization of the list of PAMIs. This approach allows for the inclusion of local knowledge and expertise of the subnational teams to capture the unique needs of different regions.

## Limitations

The Ministry of Health surveillance database mainly captures suspected cases and deaths that were reported at health facilities. It may have missed community cases and deaths, leading to an underestimation of the cholera burden. The routine surveillance data is prone to suboptimal quality and completeness. It was impossible to differentiate between zero cases reported versus missing reports, hence the possibility that the priority index and laboratory testing were underestimated for some areas. There was a lack of recent and reliable data at the subnational level for some vulnerability factors, limiting the application of these factors to include additional areas. The Ministry of Health should push for regular surveys to include relevant vulnerability factors at the subnational level. There were a high number of sub-counties with biased indicators (due to insecurity and limited access to health facilities), leading to more areas identified as additional PAMIs. The qualitative criteria applied to the additional PAMIs are different and not comparable to the quantitative criteria used to identify initial PAMIs.
